# Zoledronic acid produces analgesia by inhibiting the transient receptor potential vanilloid 1 (TRPV1) channel

**DOI:** 10.1097/PR9.0000000000001477

**Published:** 2026-07-16

**Authors:** Karina Carvajal-Zamorano, César A. Amaya-Rodríguez, Ana Gómez del Campo, Angelina Palacios-Muñoz, Nicolas M. Ardiles, Carlos Ancatén-González, Rodrigo C. Meza, Wladimir Plaza-Briceño, Ignacio Segura, Rosa Scala, Domenico Tricarico, Pablo R. Moya, Rodolfo Madrid, Andrés E. Chávez, Ramón Latorre, Karen Castillo

**Affiliations:** a Centro Interdisciplinario de Neurociencia de Valparaíso, Facultad de Ciencias, Universidad de Valparaíso, Valparaíso, Chile; b Departamento de Fisiología y Comportamiento Animal, Universidad de Panamá, Facultad de Ciencias Naturales, Exacta y Tecnología, Ciudad de Panamá, Panamá; c Departamento de Biología, Facultad de Química y Biología, Universidad de Santiago de Chile, Santiago, Chile; d Instituto de Neurociencia, Facultad de Ciencias, Universidad de Valparaíso, Valparaíso, Chile; e Centro de Investigación en Ciencias Odontológicas y Médicas, Facultad de Odontología, Universidad de Valparaíso, Valparaíso, Chile; f Instituto de Fisiología, Universidad de Valparaíso, Valparaíso, Chile; g Department of Cell Biology and Physiology and the Center for Investigation of Membrane Excitability Diseases, Washington University School of Medicine, St Louis, MO, USA; h Section of Pharmacology, Department of Pharmacy-Pharmaceutical Sciences, University of Bari, Bari, Italy

**Keywords:** TRPV1, Zoledronic acid, Pain, Ion channel, Pore block, Nociception, Bisphosphonates

## Abstract

**Introduction:**

The TRPV1 channel is activated by noxious heat and capsaicin and is expressed in primary nociceptive fibers, where it plays a key role in pain perception and inflammation. Although vanilloid compounds can induce topical analgesia, their systemic use is limited by thermoregulatory side effects. Zoledronic acid (ZOL), a bisphosphonate widely used to treat bone disorders and cancer-associated bone loss, has reported analgesic properties, although its effects on TRPV1 remain incompletely understood.

**Objective:**

To determine whether ZOL modulates TRPV1 channel function and to elucidate the mechanism underlying its analgesic effects.

**Methods:**

We combined behavioral assays of thermal nociception in mice and *Drosophila melanogaster*, Ca^2+^ imaging in primary dorsal root ganglion neurons, electrophysiological recordings from TRPV1 expressed in *Xenopus laevis* oocytes, and acute hippocampal slice recordings to assess TRPV1-dependent synaptic plasticity.

**Results:**

Systemic ZOL administration significantly reduced thermal nociceptive behaviors in both models without affecting locomotion or core temperature. In vitro, ZOL decreased capsaicin-evoked Ca^2+^ responses in dorsal root ganglion neurons and markedly reduced TRPV1-mediated currents in excised membrane patches from oocytes. In hippocampal slices, ZOL prevented TRPV1-dependent synaptic depression without altering basal transmission. Loss of inhibition under asymmetric ionic strength conditions suggests a pore-blocking mechanism.

**Conclusions:**

Our findings identify a previously unrecognized pore-blocking mechanism by which zoledronic acid inhibits TRPV1 channel activity, providing a mechanistic explanation for its analgesic effects. These results support a potential role for bisphosphonates as scaffolds for the development of novel analgesics and as tools to probe TRPV1-mediated pain pathways.

## Abstract 2

Zoledronic acid inhibits TRPV1 function, reduces nociceptive responses in preclinical models, and reveals a mechanistic basis for analgesia with potential clinical repurposing.

## 1. Introduction

Pain is the leading cause of medical consultation and is associates with reduced autonomy, decreased quality of life, and substantial economic costs.^4,10^ Together with the dose-limiting adverse effects of opioids and NSAIDs, and the risk of dependence associated with prolonged opioid use, this highlights the need for new analgesic strategies. The TRPV1 ion channel is a key target in pain pathways,^7^ integrating nociceptive and inflammatory signals.^6,7^ It is expressed in nociceptive neurons of the dorsal root and trigeminal ganglia, as well as in peripheral and central tissues, where its activation contributes to thermal hyperalgesia, pruritus, neuropathic pain, and inflammation.^6,27^

TRPV1 is a polymodal channel activated by noxious heat (≥42°C), low pH (6.0), inflammatory mediators, endogenous lipids, toxins, and vanilloids such as capsaicin (CAP).^7,27,36^

Owing to its key role in pain, TRPV1 has been widely pursued as an analgesic target.^2^ Although agonists such as CAP can produce analgesia through nociceptor desensitization, their clinical use is often limited by poor tolerability. Consequently, TRPV1 antagonism remains a promising alternative, as several antagonists have shown analgesic efficacy in preclinical models and demonstrated antinociceptive effects in clinical trials.^16,34,44,49^ However, their systemic administration has been associated with thermoregulatory side effects, including hyperthermia, limiting clinical translation.^5,14,17,25^

Zoledronic acid (ZOL), a nitrogen-containing bisphosphonate, is widely used to treat bone disorders by reducing bone resorption, preserving bone structure, and lowering fracture risk.^39^ In addition to its skeletal effects, ZOL and other bisphosphonates (BPs) have been reported to exert antinociceptive, antiallodynic, and antihyperalgesic effects across multiple pain models and in patients with bone metastases,^45^ although the underlying mechanisms remain poorly understood.^12,40^ Notably, our group previously reported that ZOL can activate TRPV1-dependent currents.^44^ This finding contrasts with the analgesic effects observed in vivo and raises the possibility that ZOL–TRPV1 interactions may depend on experimental conditions. In particular, differences in the ionic environment may critically influence the functional effects of ZOL on TRPV1 activity. These observations prompted us to re-examine the mechanism of ZOL action on TRPV1 under controlled experimental conditions. Here, we show that ZOL inhibits TRPV1 channel activity through a mechanism consistent with pore occlusion, providing a mechanistic framework that reconciles these apparently divergent observations.

TRPV1 is highly expressed in bone-innervating nociceptors and activated by the acidic and thermal microenvironments associated with bone pain,^45^ supporting the possibility that ZOL exerts analgesic effects through TRPV1 modulation. The present work shows that ZOL robustly suppresses TRPV1-mediated nociceptive responses in mice and *Drosophila melanogaster*, as well as TRPV1-mediated Ca^2+^ signals in primary sensory neurons and channel activity recorded in *Xenopus laevis* oocytes and acute mouse brain slices.

## 2. Methods

### 2.1. Ethical approval

All animal procedures complied with institutional and national guidelines and were approved by the CICUAL (Universidad de Valparaíso) and the Bioethical Committee of Universidad de Santiago de Chile (Protocols BEA 199-23, CBC 88-2023, CBC 124-2024, BEA 219-25, and 619-2025).

### 2.2. Mice behavioral assays

Male and female wild-type C57BL/6J mice (2–3 months old, 20–25*g*) received intraperitoneal injections of saline (0.9% NaCl, 5 mL/kg), ZOL (100 µg/kg), or AMG9810 (30 mg/kg). Thermal sensitivity was assessed using the hot plate test (45, 50, 55°C), recording latency to nocifensive behaviors (hind paw licking or jumping). Cut-off times were set at 90 seconds, 60 seconds, and 40 seconds, respectively, to prevent tissue damage. CAP-induced nociception was evaluated by measuring paw-licking behavior after intraplantar injection (10 µL) of vehicle, CAP (0.75 µg), ZOL (3.5 µg), or CAP + ZOL. Behavioral responses were recorded and analyzed using Ethovision Video Tracking System and ANY-maze.

To assess thermoregulation, rectal temperature was measured in a separate cohort at baseline and up to 24 hours after systemic administration of saline or ZOL treatment.

### 2.3. *Drosophila* thermal avoidance

Wild-type *white*^*1118*^ (*w*^*1118*^) flies (10–15 days old) and transgenic flies expressing human TRPV1 under pain-GAL4 control (BL27894, Bloomington; UAS-hTRPV1 from KDRC)^26^ were maintained on standard cornmeal medium at 20 to 22°C under a 12-hour light:12-hou dark cycle. Flies were fed zoledronic acid (ZOL, 100 nM-300 µM) or AMG9810 (100 nM-100 µM) for 5 days before testing. Thermal nociception was evaluated in control flies (pain-GAL4>w1118, control A; UAS-hTRPV1>w1118, control B) and flies expressing human TRPV1 (pain-GAL4>UAS-hTRPV1, control AB) in which flies were exposed to a 46°C water-bath paradigm for 4 minutes. To control potential phototaxis, a light source was positioned beneath the apparatus during testing. Avoidance behavior was quantified as the percentage of flies avoiding contact with the heated surface.

### 2.4. Dorsal root ganglion culture and Ca^2+^ imaging

Primary dorsal root ganglion (DRG) neurons from P30–P90 C57BL/6 mice were dissociated and cultured as previously described.^11,18^ Ganglia were enzymatically treated with collagenase XI (650 U/mL) and dispase (5 U/mL) prepared in INC-mix solution (in mM: NaCl 155, K_2_HPO_4_ 1.5, HEPES 10, Glucose 5, pH 7.4) for 40 minutes at 37°C, mechanically dissociated, and plated on poly-l-lysine–coated glass coverslips. Cells were maintained in MEM supplemented with 10% FBS and penicillin and used for Ca^2+^ imaging 6 to 12 hours after plating.

Ratiometric Ca^2+^ imaging was performed using Fura-2 AM (5 µM) as previously described.^35^ Fluorescence (340/380 nm excitation, 510 nm emission) was acquired at 0.5 Hz on an inverted microscope and recorded using HCImage software. Bath temperature was monitored with a thermocouple system and maintained at 34 ± 1°C. Cells were continuously perfused with extracellular solution (in mM: 140 NaCl, 3 KCl, 1.3 MgCl_2_, 2.4 CaCl_2_, 10 HEPES, 10 glucose; 298 mOsm/kg, pH 7.4).

TRPV1 responses were evoked using a double-pulse protocol with capsaicin (20 nM). ZOL (10 µM) or AMG9810 (1 µM) were applied 3 minutes before and during the second pulse.

### 2.5. Electrophysiology in *X. laevis* oocytes

*X*. *laevis* oocytes were used for heterologous expression of wild-type TRPV1 (GenBank NM031982). cRNA was synthesized in vitro and 150 ng were injected per oocyte. Oocytes were incubated in ND96 solution (in mM: 96 NaCl, 2 KCl, 1.8 CaCl_2_, 1 MgCl_2_, 5 HEPES, pH 7.4) at 18°C for 3 to 5 days before recordings.

TRPV1 currents were recorded using patch-clamp in inside-out and outside-out configurations. Symmetrical recording solutions contained (in mM): 150 NaCl, 10 EGTA, 2 MgCl_2_, 10 HEPES, pH 7.4; pore unblock experiments used Na^+^-reduced solution (10 NaCl, 140 NMDG). ZOL (3 mM) and CAP (50 mM) stock solutions were prepared in Milli-Q water and absolute ethanol, respectively, and diluted to final concentrations in recording solutions before perfusion.

Currents were recorded using an Axopatch 200B amplifier and Clampex 10.7 software. Signals were acquired at 100 kHz, filtered at 20 kHz (8-pole Bessel), and digitized with a 16-bit A/D converter (Digidata 1550B). Experiments were performed at room temperature (18–20°C). Macroscopic currents were evoked by voltage steps from −60 to +260/+300 mV and then returned to −60 mV. For single-channel recordings, patches were held at constant membrane potentials for 2 minutes.

### 2.6. Hippocampal slices and electrophysiological recordings

Acute hippocampal slices (350 µm) from P20-P35 mice were prepared as previously described,^8^ in a solution containing (in mM): 215 sucrose, 2.5 KCl, 26 NaHCO_3_, 1.6 NaH_2_PO_4_, 1 CaCl_2_, 4 MgCl_2_, 4 MgSO_4_, and 20 glucose. Thirty minutes postsectioning, the cutting solution was gradually replaced with an artificial cerebrospinal fluid (ACSF) recording solution containing (in mM): 124 NaCl, 2.5 KCl, 26 NaHCO_3_, 1 NaH_2_PO_4_, 2.5 CaCl_2_, 1.3 MgSO_4_, and 10 glucose. All solutions were equilibrated with 95%O_2_/5% CO_2_ (pH 7.4), and slices were incubated in ACSF for at least 60 minutes before recordings.

Whole-cell recordings were obtained from dentate granule cells held voltage clamped at −60 mV (Multiclamp 700A) at 28 ± 1°C in ACSF containing picrotoxin (100 µM). Patch electrodes (3–4 MΩ) were filled with internal solution (in mM: 131 Cs-gluconate, 8 NaCl, 1 CaCl_2_, 10 EGTA, 10 glucose, 10 HEPES; pH 7.2, 285 mOsm/kg). Series resistance was monitored, and cells with >20% change were excluded.

Excitatory synaptic responses were evoked by stimulating medial perforant path (MPP) inputs. TRPV1-dependent LTD was induced using a pairing protocol (70 milliseconds interval) combined with postsynaptic depolarization (30 mV, 30 milliseconds), repeated 900 times at 1 Hz.^8^

CAP, capsazepine (CPZ), and ZOL were bath-applied after a stable baseline (10–15 minutes). Cells with >10% rundown were excluded. EPSCs were filtered at 2.2 kHz and acquired at 5 kHz.

### 2.7. Data analysis and statistics

Data are presented as mean ± SEM. Behavioral data were analyzed using 2-way ANOVA followed by the Tukey post hoc test. Nonparametric data were analyzed using Kruskal–Wallis tests with Dunn post hoc comparisons. Electrophysiological data were normalized to control responses when appropriate. Statistical significance was set at *P* < 0.05. Analyses were performed in GraphPad Prism 10.0 (GraphPad Software, CA), and representative traces were plotted using OriginPro 8.6.

### 2.8. Materials

Capsaicin, capsazepine, and collagenase XI were from Sigma-Aldrich. Zoledronic acid (6111) and picrotoxin were from Tocris Bioscience, and AMG9810 from AK Scientific. Dispase (17105–041), MEM medium and supplements, streptomycin/penicillin, Fura-2 AM, and Pluronic F-127 were from Thermo Fisher Scientific. Fetal bovine serum was from HyClone.

CAP was dissolved in ethanol or DMSO, AMG9810 and picrotoxin in DMSO, and ZOL in water. Stock solutions were freshly diluted; final solvent concentrations did not exceed 0.01%.

## 3. Results

### 3.1. Zoledronic acid reduces nociceptive behavior in mice

**Figure 1.**
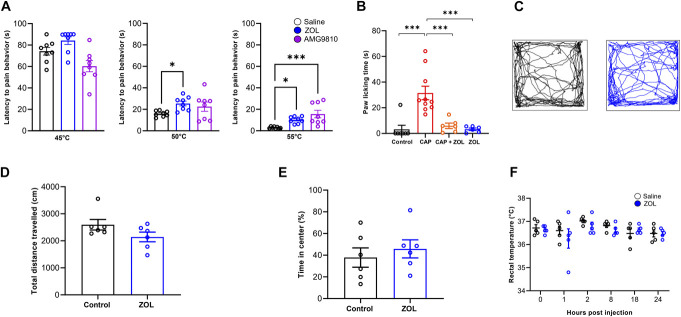
Zoledronic acid reduces TRPV1-mediated nociceptive responses without affecting locomotor activity or thermoregulation. (A) Acute administration of ZOL (100 μg/kg, i.p.) increased withdrawal latency in the hot plate test at 50°C and 55°C, mimicking the effect of the antagonist AMG9810 (30 mg/kg). Data are presented as mean ± SEM with individual data points (n = 8 per group). (B) Capsaicin (CAP)-induced paw-licking behavior was significantly reduced by ZOL (5.97 ± 2.10 seconds; n = 6) compared with CAP alone (31.52 ± 5.28 seconds; n = 10), reaching values similar to control groups (vehicle = 3.17 ± 3.17 seconds; ZOL alone = 3.25 ± 0.96 seconds; n = 6; 2-way ANOVA, CAP and ZOL ***P < 0.0002). (C–E) Open field parameters, including total distance traveled (C), number of center entries (D), and time spent in the center (E), were not affected by ZOL treatment, indicating no changes in locomotor or anxiety-like behavior. Data are expressed as mean ± SEM, n = 6 per group. (F) Rectal temperature measurements after acute ZOL administration (100 µg/kg, i.p.) revealed no significant differences compared with saline-treated mice at any time point (2-way ANOVA; n = 5 per group), indicating no detectable effects on thermoregulation.

Mice received a single intraperitoneal injection of ZOL (100 µg/kg, i.p.)^22^ or saline and were subsequently placed on a metal surface maintained at 45°, 50°, or 55°C (Fig. 1A). Latency to nocifensive behaviors (hind paw licking or jumping) was used as an index of thermal sensitivity. ZOL did not modify responses at 45°C (Fig. 1A, left panel), but significantly increased response latencies at 50°C and 55°C compared with saline-treated mice (Fig. 1A, middle and right panels). The magnitude of this effect was comparable with that produced by the selective TRPV1 antagonist AMG9810 (30 mg/kg). In the capsaicin-induced paw-licking assay, ZOL markedly reduced nocifensive behavior (5.97 ± 2.10 seconds; n = 6) compared with the CAP group (31.52 ± 5.28 seconds; n = 10), reaching values similar to vehicle and ZOL-alone groups (3.17 ± 3.17 seconds and 3.25 ± 0.96 seconds, respectively; Fig. 1B). To exclude confounding effects on locomotion or anxiety-like behavior, mice were evaluated in the open field test. Zoledronic acid did not affect total distance traveled, number of center entries (Figs. 1C–D), or time spent in the center (Fig. 1E), indicating that the antinociceptive effects were not secondary to sedation or motor impairment.

Given the known thermoregulatory effects associated with TRPV1 antagonists as well as agonists that induce desensitization such as CAP, we next evaluated whether ZOL alters core body temperature under the same dosing conditions. Rectal temperature measurements revealed no significant differences between ZOL- and saline-treated mice at any time point (Fig. 1F), indicating that the observed behavioral effects are not accompanied by detectable thermoregulatory alterations.

### 3.2. Zoledronic acid reduces TRPV1-dependent nociceptive behavior in *Drosophila*

*Drosophila melanogaster* is a powerful model for nociception due to well-characterized sensory neurons, conserved ascending and descending pain pathways, and strong evolutionary conservation of pain mechanisms.^19^ Transgenic flies expressing human pain receptors enable pharmacological testing with clinically relevant compounds.^26^ Notably, expression of human TRPV1 in flies mediates robust CAP-induced nociception that can be modulated by NSAIDs, gabapentin, and morphine, underscoring the model's translational value.

**Figure 2.**
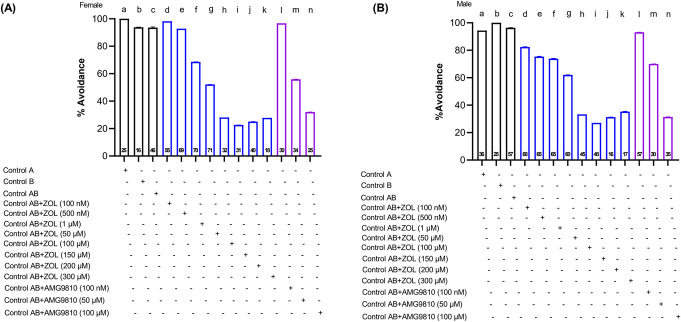
Zoledronic acid reduced TRPV1-dependent nociceptive behavior in *Drosophila*. (A) Female *Drosophila melanogaster* expressing human TRPV1 in sensory nociceptor neurons (pain-GAL4>UAS-hTRPV1, control AB) displayed robust avoidance to a 46°C thermal stimulus under control conditions (∼100%). Dietary exposure to ZOL for 5 days induced a dose-dependent reduction in avoidance, with significant effects at concentrations ≥150 µM, comparable with those observed with the TRPV1 antagonist AMG9810. Control genotypes (*pain-GAL4>w1118*, control A; *UAS-hTRPV1>w1118*, control B) showed normal avoidance behavior. (B) Male flies expressing human TRPV1 exhibited a similar dose-dependent decrease in avoidance after ZOL or AMG9810 treatment, whereas all control groups maintained high avoidance. Distinct letters above bars indicate statistically significant differences among groups.

To examine ZOL-TRPV1 interaction in vivo, we used a thermal avoidance assay in *D. melanogaster* expressing human TRPV1 in nociceptive neurons under the pain-GAL4 driver, which targets multidendritic neurons innervating most of the body, including mouthparts and the antennal–maxillary complex.^26,32^ Control genotypes (pain-GAL4>w1118, control A; UAS-hTRPV1>w1118, control B; pain-GAL4>UAS-hTRPV1, control AB) showed robust avoidance to 46°C (∼100%) (Fig. 2A). After 5 days of dietary ZOL, pain-GAL4>UAS-hTRPV1 flies displayed dose-dependent reduction in avoidance, significant at ≥150 μM (∼20–40%), comparable with AMG9810. Males showed the same pattern, with strong baseline avoidance and progressive suppression in ZOL- and AMG9810-fed groups (Fig. 2B). These results demonstrate dose-dependent suppression of TRPV1-mediated nociception by ZOL in both sexes, consistent with channel inhibition.

### 3.3. Zoledronic acid reduces capsaicin-evoked calcium responses in primary sensory neurons

**Figure 3.**
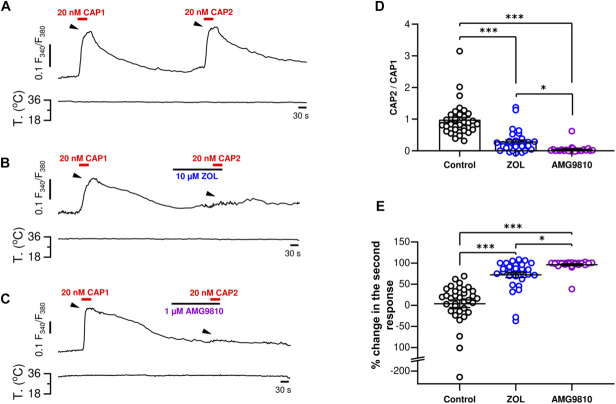
Zoledronic acid reduces capsaicin-evoked calcium responses in primary sensory neurons. (A–C) Representative Fura-2 ratiometric traces of intracellular calcium ([Ca^2+^]_i_) recorded from cultured dorsal root ganglion (DRG) neurons in response to 2 consecutive applications of capsaicin (CAP, 20 nM), under control conditions (A) or in the presence of zoledronic acid (ZOL, 10 µM; B) or the selective TRPV1 antagonist AMG9810 (1 µM; C). Bath temperature was recorded simultaneously (lower panels). Arrowheads indicate the peak of each CAP-evoked response. (D) Ratio of the second to the first response (CAP2/CAP1) for individual neurons under each condition. Each point represents a single neuron. (E) Quantification of the normalized change in the second CAP-evoked response relative to the first. Both ZOL and AMG9810 significantly reduced TRPV1-mediated calcium signals. Data are presented as mean ± SEM. Statistical comparisons were performed using a Kruskal–Wallis test followed by the Dunn multiple comparisons test (***P < 0.0001, *P < 0.05; control, n = 34, zoledronic acid, n = 30, and AMG9810, n = 26 neurons).

To determine whether ZOL directly modulates TRPV1 activity in primary somatosensory neurons, we monitored capsaicin (CAP)-induced calcium dynamics in cultured DRG neurons using Fura-2 ratiometric Ca^2+^ imaging. Under control conditions, 2 consecutive applications of a subsaturating concentration of CAP (20 nM) evoked reproducible [Ca^2+^]_i_ transients of similar amplitude (Fig. 3A). By contrast, exposure to ZOL (10 µM) markedly reduced the second response to CAP (Fig. 3B), an effect comparable with that observed with the selective TRPV1 antagonist AMG9810 (1 µM; Fig. 3C). Quantitative analysis of the CAP2/CAP1 ratio revealed a significant decrease in ZOL- and AMG9810-treated neurons relative to control cells (Fig. 3D). Consistently, the normalized change in the second CAP-evoked Ca^2+^ response was significantly reduced by both treatments, with an average decrease of 72% and 96% in the ZOL and AMG9810 groups, respectively (Fig. 3E). Together, these results indicate that ZOL suppresses CAP-evoked Ca^2+^ influx in primary DRG neurons, consistent with functional inhibition of TRPV1 channels.

### 3.4. Zoledronic acid directly inhibits TRPV1 channel activity in excised membrane patches

**Figure 4.**
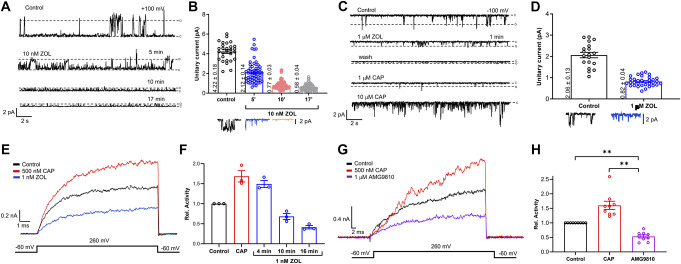
Zoledronic acid reduces capsaicin- and voltage-activated TRPV1 currents in excised membrane patches from *X. laevis* oocytes. (A) Representative single-channel recordings of TRPV1 at +100 mV in the inside-out configuration showing progressive inhibition after exposure to ZOL (10 nM) at 5, 10, and 17 minutes. “C” and “O” denote closed and open channel states, respectively. (B) Unitary conductance decreases from ∼42 pS (control) to ∼21 pS (5 minutes), ∼7 pS (10 minutes), and ∼5.8 pS after 17 minutes (n = 5–7). Bottom panels show zoomed-in segments illustrating conductance reduction. (C–D) Representative TRPV1 single-channel recordings at −100 mV under control conditions and after perfusion with ZOL (1 μM), showing a decrease in conductance from 2.06 ± 0.13 pS to 0.82 ± 0.04 pS (n = 5). Activity was not recovered after washout but was restored after exposure to 10 µM CAP. Bottom panels show representative traces of single-channel activity under control (left) and ZOL-treated (right) conditions. (E) Representative macroscopic TRPV1 currents recorded in inside-out configuration using a voltage step protocol (−60 mV to +260 mV). Application of 500 nM CAP (red trace) potentiated TRPV1 currents, whereas subsequent ZOL exposure (light blue trace) progressively reduced current amplitude. (F) Bar plot of normalized current activity under control, CAP, and ZOL conditions (n = 3). (G) Representative macroscopic TRPV1 currents showing inhibition by AMG9810 (1 µM, purple trace) after CAP activation. (H) Normalized current activity under control, CAP, and AMG9810 conditions (n = 9). Data are presented as mean ± SEM.

To determine whether ZOL directly modulates TRPV1 activity, single-channel recordings were obtained from excised inside-out patches of *X. laevis* oocytes expressing TRPV1. At +100 mV, ZOL (10 nM) progressively decreased channel activity, producing a stepwise reduction in unitary conductance from ∼42 pS (control) to ∼21 pS (5 minutes), ∼7 pS (10 minutes), and ∼5.8 pS after 17 minutes (n = 5–7) (Figs. 4A, B). At −100 mV, 1 µM ZOL reduced conductance from 2.06 ± 0.13 pS to 0.82 ± 0.04 pS (n = 5), with no recovery after washout; activity was only partially restored by CAP (10 µM) (Figs. 4C, D).

Macroscopic inside-out recordings confirmed this inhibition: CAP (500 nM) strongly potentiated TRPV1 currents, whereas subsequent ZOL exposure caused a marked progressive decrease in current amplitude (Figs. 4E, F). This effect was comparable with inhibition by the selective TRPV1 antagonist AMG9810 (1 µM) (Figs. 4G, H). Together, these results show that ZOL directly inhibits TRPV1 in excised patches by reducing unitary conductance and open probability, with an inhibitory profile resembling that of AMG9810.

### 3.5. Zoledronic acid inhibits TRPV1 channels from both membrane sides with preference for the open state

**Figure 5.**
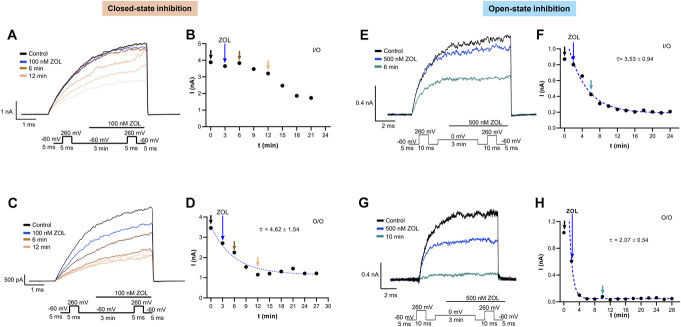
Zoledronic acid inhibits TRPV1 channel activity from both intracellular and extracellular membrane surfaces with a preference for the open state. Top row: TRPV1 inhibition from the closed state. (A–B) Representative macroscopic TRPV1 currents recorded in inside-out (I/O, A) and outside-out (O/O, B) patch configurations. Membrane patches were held at −60 mV and briefly depolarized (5 milliseconds, +260 mV) to assess channel activity. ZOL (100 nM) was applied during a prolonged holding potential at −60 mV (3 minutes), a condition in which channels remain predominantly closed. A final depolarizing pulse was then applied to evaluate channel activity. (C–D) Normalized current amplitudes under these conditions show that ZOL produced a slow and incomplete inhibition of TRPV1 currents. In the O/O configuration, the inhibition could be fitted with a monoexponential decay function (τ = 4.82 ± 1.54 minutes), whereas in the I/O configuration, the time course did not follow a simple exponential behavior. Bottom row: TRPV1 inhibition from the open state. (E–F) Representative TRPV1 currents recorded in I/O and O/O configurations with membrane patches held at 0 mV, a condition that favors channel opening. Application of ZOL (3 minutes) produced a clear time-dependent inhibition of TRPV1 currents in both configurations. (G–H) Normalized current amplitudes show that ZOL induced a robust monoexponential decay of current, with time constants (τ) of 3.53 ± 0.9 minutes (I/O) and 2.07 ± 0.54 minutes (O/O).

Zoledronic acid reduced TRPV1 currents when applied to both the intracellular (inside-out, I/O) and extracellular (outside-out, O/O) membrane surfaces (Fig. 5), indicating that it can access its inhibitory site from either side of the membrane. Representative macroscopic recordings from I/O and O/O configurations showed that when ZOL (100 nM) was applied during a prolonged holding potential at −60 mV, a condition in which channels remain predominantly closed (Figs. 5A, B), TRPV1 currents progressively declined (Figs. 5C, D). Under these conditions, the time course of inhibition could not be fitted to a single-exponential function, suggesting a slow or heterogeneous blocking process. By contrast, when ZOL (500 nM) was applied while the membrane was held at 0 mV, a condition that favors channel opening (Figs. 5E, F), inhibition developed progressively and was well-described by a monoexponential decay. The mean time constants (τ) were 3.53 ± 0.9 minutes for the I/O configuration (Figs. 5G) and 2.07 ± 0.54 minutes for the O/O configuration (Fig. 5H). These results indicate that ZOL inhibits TRPV1 from both membrane sides, with faster inhibitory kinetics when channels are in the open state, consistent with state-dependent inhibition.

### 3.6. Zoledronic acid inhibits TRPV1 currents by reversible pore occlusion under high electrochemical driving force

**Figure 6.**
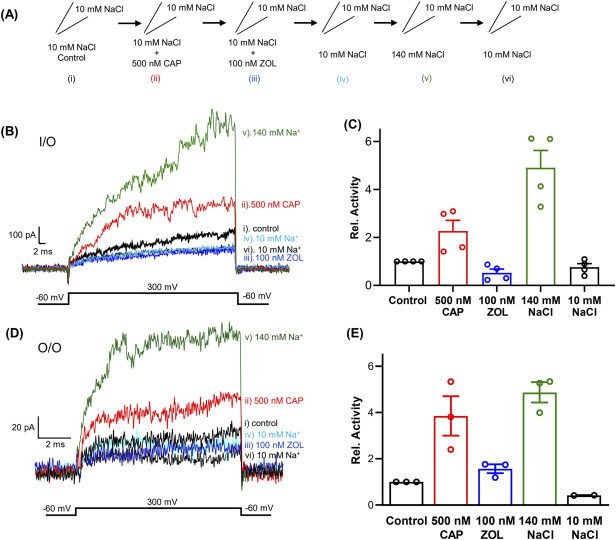
Zoledronic acid inhibits TRPV1 by occluding the ion permeation pathway. (A) Voltage-step protocol (−60 mV to +300 mV) used to evoke TRPV1 macroscopic currents under the indicated conditions. (B–C) Representative recordings of TRPV1 currents in inside-out (I/O, B) and outside-out (O/O, C) configurations. Under control conditions (trace i), TRPV1 activity was potentiated by capsaicin (CAP, 500 nM; trace ii). Application of zoledronic acid (ZOL, 100 nM; trace iii) markedly reduced current amplitude, and inhibition persisted after washout under low ionic strength conditions (trace iv). Increasing the external NaCl concentration to 140 mM, while maintaining CAP and ZOL, resulted in a substantial recovery of current amplitude (trace v). Returning to low ionic strength (10 mM NaCl) in the continued presence of CAP and ZOL reinstated the inhibition (trace vi). (D–E) Bar plots showing normalized TRPV1 current activity under control (black), CAP (red), CAP + ZOL (blue), and CAP + ZOL in high NaCl (green/black) conditions in I/O (n = 4, D) and O/O (n = 3, E) recordings. Data are presented as mean ± SEM.

Given that ZOL inhibited TRPV1 from either membrane side, we examined whether it directly occludes the ion permeation pathway. Macroscopic TRPV1 currents were recorded under low ionic strength (symmetrical 10 mM NaCl) using voltage steps from −60 mV to +300 mV (Fig. 6A). In both I/O and O/O configurations, CAP (500 nM) strongly potentiated currents (trace ii) compared with control (trace i), whereas subsequent application of ZOL (100 nM) produced marked inhibition (trace iii) that persisted after washout under low ionic strength conditions (trace iv) (Figs. 6B, C). Increasing the external NaCl concentration to 140 mM, while maintaining CAP and ZOL, resulted in a substantial recovery of current amplitude (trace v). When the ionic strength was returned to 10 mM NaCl, the current decreased again toward inhibited levels (trace vi). This reversible modulation indicates that elevated ionic strength weakens ZOL-mediated inhibition, consistent with electrostatic displacement and reduced occupancy of ZOL within the pore due to increased electrochemical driving force. Normalized current analysis confirmed significant TRPV1 inhibition by ZOL in both I/O (n = 4) and O/O (n = 3) configurations, with partial recovery on increasing NaCl concentration (Figs. 6D, E). Together, these results support a pore-blocking mechanism in which ZOL interacts with the conduction pathway, producing reversible inhibition modulated by ionic strength.

### 3.7. Zoledronic acid prevents TRPV1-dependent synaptic depression without affecting basal transmission

**Figure 7.**
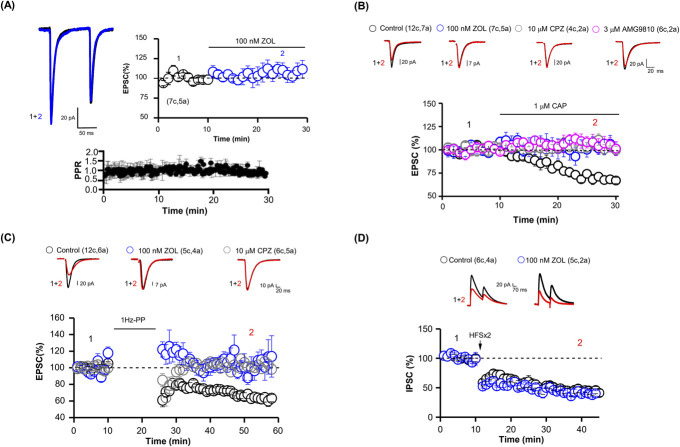
Zoledronic acid prevents TRPV1-dependent synaptic depression without affecting basal synaptic transmission. (A) Representative trace of excitatory postsynaptic currents (EPSCs), recorded from dentate granule cells (DGCs) and elicited at the medial perforant pathway (MPP-DGC) synapses. Application of ZOL (100 nM) did not alter either EPSC amplitude or paired-pulse ratio (PPR), indicating no effect on basal synaptic transmission or presynaptic release probability. (B) Capsaicin (CAP, 1 µM)-induced depression of AMPAR-mediated EPSCs was abolished in the presence of ZOL (100 nM), capsazepine (CPZ, 10 µM), or AMG9810 (3 µM), confirming inhibition of TRPV1-dependent effects. (C) Low-frequency stimulation (1 Hz)-induced TRPV1-dependent long-term depression (LTD) was prevented by ZOL and CPZ. (D) CB1 receptor-dependent inhibitory plasticity was unaffected by ZOL, indicating selectivity for TRPV1-dependent processes. Representative traces are shown above each plot. Alphanumeric labels (eg, “12c, 7a”) indicate the number of cells (C) and animals (A) contributing to each data set and are used to identify trace correspondence within each panel. Data are presented as mean ± SEM.

TRPV1 has been implicated in synaptic function and plasticity in several brain regions, including the hippocampus.^8^ To evaluate whether ZOL modulates TRPV1-dependent synaptic activity in a native neuronal context, we examined its effects on excitatory synaptic transmission in hippocampal slices. This preparation was used as a well-established functional assay of TRPV1-dependent synaptic modulation, rather than to assess blood–brain barrier permeability or pain-related circuitry. EPSCs were recorded at medial perforant pathway-dentate granule cell (MPP-DGC) synapses (Fig. 7A). CAP (1 µM) induced a robust depression of AMPAR-mediated EPSCs, confirming the TRPV1 dependence of this synaptic response, which was prevented by the TRPV1 antagonists capsazepine CPZ, (10 µM) and AMG9810 (3 µM), as well as by ZOL (100 nM) (Fig. 7B), consistent with inhibition of TRPV1-dependent synaptic modulation.

Similarly, low-frequency stimulation (1 Hz)-induced TRPV1-dependent long-term depression (LTD) was prevented in the presence of ZOL or CPZ (Fig. 7C). By contrast, CB1 receptor-dependent inhibitory plasticity^9^ was unaffected by ZOL (Fig. 7D), supporting the selectivity of its effect on TRPV1-dependent processes.

## 4. Discussion

In this study, we demonstrate that zoledronic acid (ZOL) exerts a previously unrecognized modulatory effect on TRPV1 that translates into a predominantly antihyperalgesic effect. This conclusion is supported by converging evidence across behavioral, cellular, and electrophysiological approaches. In vivo, ZOL modestly increased thermal nociceptive thresholds but markedly suppressed capsaicin-evoked nocifensive behavior, indicating a preferential effect on TRPV1-dependent sensitized states. Consistently, ZOL reduced nociceptive responses in *Drosophila* expressing human TRPV1, as well as capsaicin-evoked Ca^2+^ signals in primary DRG neurons. Electrophysiological recordings further demonstrated direct inhibition of TRPV1 channel activity from both membrane sides through a state-dependent pore-blocking mechanism. In hippocampal slices, ZOL abolished TRPV1-dependent synaptic depression without affecting basal transmission or CB1 receptor-mediated responses, supporting selective functional inhibition of TRPV1-mediated processes. Together, these findings establish ZOL as a functionally relevant TRPV1 inhibitor across multiple experimental systems.

### 4.1. TRPV1 as a validated but challenging target for analgesia

TRPV1 is activated by thermal, acidic, and chemical stimuli and contributes to pain and neurogenic inflammation through neuropeptide release (eg, CGRP and substance P). Sensitization lowers its activation threshold, enhancing pain perception and establishing TRPV1 as a key mediator of inflammatory pain and an attractive therapeutic target.

Modulators acting at the vanilloid pocket, including agonists and antagonists, can induce analgesia by promoting channel desensitization or inhibition, as exemplified by topical capsaicin and resiniferatoxin.^2^ However, systemic TRPV1 agonists cause adverse effects such as hypothermia and locomotor impairment.^15,24,25^ Early antagonists such as capsazepine showed preclinical efficacy but failed clinically due to poor bioavailability and off-target effects.^3^ Although numerous TRPV1 inhibitors have advanced to clinical trials,^1^ maintaining efficacy without disrupting thermoregulation remains a major challenge.

### 4.2. Zoledronic acid as a noncanonical TRPV1 inhibitor

In contrast to classical TRPV1 antagonists, ZOL, a third-generation nitrogen-containing bisphosphonate, has not been traditionally classified as an analgesic agent. Its clinical use in metabolic bone disease and cancer-associated bone pain has largely been attributed to inhibition of farnesyl pyrophosphate synthase (FPPS) in the mevalonate pathway.^42,43^ In addition, ZOL exhibits antiangiogenic, proapoptotic, and anti-inflammatory effects.^13,21,28,31,33,41,47^ Although ZOL alleviates bone pain in clinical settings,^30,43^ the underlying molecular mechanisms have remained unclear.^45^

The present data substantially extend this pharmacological profile by demonstrating that ZOL directly inhibits TRPV1 channel activity through a reversible pore-blocking mechanism. This effect occurs independently of its canonical actions on osteoclasts or protein prenylation and does not require covalent channel modification, suggesting a distinct mode of interaction compared with irreversible TRPV1 modulators.

Importantly, the behavioral profile observed here indicates that ZOL preferentially reduces TRPV1-dependent hypersensitivity rather than baseline nociceptive responses. This distinction is consistent with an antihyperalgesic mechanism of action and may have important therapeutic implications in conditions characterized by peripheral sensitization.

### 4.3. Reconciling previous observations of TRPV1 modulation by bisphosphonates

Initial evidence suggesting an interaction between bisphosphonates and TRPV1 arose from previous work by our group,^38^ which reported that ZOL enhanced TRPV1-dependent currents under specific experimental conditions.

The apparent discrepancy with the present findings most likely reflects differences in experimental parameters rather than a fundamental inconsistency in mechanism of action of ZOL. Variations in ionic composition, electrochemical driving force, and recording configuration are expected to influence ion channel behavior and drug–channel interactions. Furthermore, differences in drug formulation and solvent composition may also contribute to distinct functional outcomes.

Importantly, this study provides a mechanistic framework that may reconcile these observations. Our results show that ZOL inhibits TRPV1 through a reversible pore-blocking mechanism strongly influenced by ionic conditions. Under ionic conditions resembling physiological extracellular NaCl levels, ZOL consistently inhibited TRPV1 in both I/O and O/O configurations. These findings suggest that ZOL–TRPV1 interactions depend on the electrochemical environment, potentially explaining previously divergent results.

### 4.4. Peripheral selectivity and thermoregulatory implications

An important translational aspect of ZOL is its established clinical use and well-characterized safety profile in humans.^29,46^ Zoledronic acid has been reported to exhibit limited penetration into the central nervous system,^46^ which may contribute to a peripheral site of action. In this context, TRPV1 inhibition may be sufficient to account for the observed behavioral effects, particularly under conditions of peripheral sensitization. Importantly, in contrast to classical TRPV1 antagonists, which frequently induce hyperthermia in vivo, ZOL did not alter core body temperature at analgesic doses under the conditions tested. This observation is particularly relevant given that thermoregulatory disturbances have been a major limitation in the clinical development of TRPV1 antagonists. While the mechanism underlying TRPV1-mediated thermoregulation remains incompletely understood, the absence of detectable temperature changes in our study suggests that ZOL may avoid this key limitation.

The hippocampal experiments included in this study should therefore be interpreted as a functional validation of TRPV1 inhibition in a native neuronal context, rather than as evidence of a central mechanism of analgesia.

### 4.5. Analgesia and bone biology: a dual therapeutic opportunity

Beyond nociception, TRPV1 is expressed in bone-resident cells and has been implicated in osteoclastogenesis and osteoblast function.^20,23^ Its dysregulation has been associated with bone-related pathologies, including osteoporosis and osteoarthritis,^39,48^ and TRPV1 inhibition has been linked to protective effects against bone loss.^23,37^

These observations raise the possibility that ZOL may exert dual therapeutic actions by simultaneously modulating bone remodeling and TRPV1-dependent pain signaling. This combined activity could be particularly advantageous in clinical contexts such as cancer-associated bone pain or osteoporotic fractures.

### 4.6. Limitations and future directions

Although ZOL showed robust antinociceptive effects, some limitations remain. Pharmacokinetic data in sensory tissues are lacking, and its high bone affinity may affect neuronal availability. The proposed pore-blocking mechanism requires structural confirmation, and the impact of peripheral TRPV1 inhibition on thermoregulation should be further evaluated.

### 4.7. Conclusion

In summary, our study identifies zoledronic acid as a noncanonical inhibitor of TRPV1 that acts through a reversible, state-dependent pore-blocking mechanism. By preferentially suppressing TRPV1-dependent hypersensitivity without affecting thermoregulation, ZOL may overcome key limitations associated with classical TRPV1-targeted therapies. These findings provide a mechanistic basis for the analgesic effects of bisphosphonates and highlight their potential as both therapeutic agents and molecular tools to investigate TRPV1 function.

## Disclosures

The authors have no conflict of interest to declare.
